# Bis[2-amino-6-methyl­pyrimidin-4(1*H*)-one-κ^2^ *N* ^3^,*O*]dichloridocadmium(II)

**DOI:** 10.1107/S1600536810035051

**Published:** 2010-09-08

**Authors:** Kamel Kaabi, Meher El Glaoui, P. S Pereira Silva, M. Ramos Silva, Cherif Ben Nasr

**Affiliations:** a Laboratoire de Chimie des Matériaux, Faculté des Sciences de Bizerte, 7021 Zarzouna, Tunisia; b CEMDRX, Physics Department, University of Coimbra, P-3004-516 Coimbra, Portugal

## Abstract

In the title compound, [CdCl_2_(C_5_H_7_N_3_O)_2_], the Cd^II^ atom is six-coordinated by two heterocyclic N atoms [Cd—N = 2.261 (2) and 2.286 (2) Å] and two O atoms [Cd—O = 2.624 (2) and 2.692 (2) Å] from two bidentate chelate 2-amino-6-methyl­pyrimidin-4(1*H*)-one ligands and two chloride ions [Cd—Cl = 2.4674 (6) and 2.4893 (7) Å]. The crystal packing is characterized by an open-framework architecture with the crystal packing stabilized by inter­molecular N—H⋯Cl and N—H⋯O hydrogen bonds.

## Related literature

For common applications of materials with open framework structures, see: Yaghi *et al.* (2003 ▶); Kitagawa *et al.* (2004 ▶). For literature on metal-organic compounds, see: Kaabi *et al.* (2010 ▶). For a discussion of geometrical features in related structures, see: Min *et al.* (2009 ▶); Qing-Yan & Li (2005 ▶); Moloto *et al.* (2003 ▶).
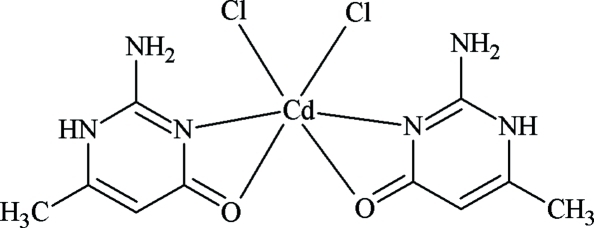


## Experimental

### 

#### Crystal data

[CdCl_2_(C_5_H_7_N_3_O)_2_]
*M* *_r_* = 433.57
Monoclinic, 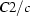

*a* = 17.4204 (5) Å
*b* = 7.5467 (2) Å
*c* = 25.4422 (6) Å
β = 106.1333 (11)°
*V* = 3213.07 (15) Å^3^
*Z* = 8
Mo *K*α radiation
μ = 1.70 mm^−1^
*T* = 293 K
0.32 × 0.20 × 0.13 mm

#### Data collection

Bruker APEXII CCD area-detector diffractometer
Absorption correction: multi-scan (*SADABS*; Sheldrick, 2003 ▶) *T*_min_ = 0.676, *T*_max_ = 0.801
41891 measured reflections
4494 independent reflections
3924 reflections with *I* > 2σ(*I*)
*R*_int_ = 0.033

#### Refinement

*R*[*F* ^2^ > 2σ(*F* ^2^)] = 0.023
*wR*(*F* ^2^) = 0.108
*S* = 1.27
4494 reflections
190 parameters
H-atom parameters constrained
Δρ_max_ = 0.74 e Å^−3^
Δρ_min_ = −1.06 e Å^−3^

### 

Data collection: *APEX2* (Bruker, 2003 ▶); cell refinement: *SAINT* (Bruker, 2003 ▶); data reduction: *SAINT*; program(s) used to solve structure: *SHELXS97* (Sheldrick, 2008 ▶); program(s) used to refine structure: *SHELXL97* (Sheldrick, 2008 ▶); molecular graphics: *PLATON* (Spek, 2009 ▶); software used to prepare material for publication: *SHELXL97*.

## Supplementary Material

Crystal structure: contains datablocks global, I. DOI: 10.1107/S1600536810035051/zs2058sup1.cif

Structure factors: contains datablocks I. DOI: 10.1107/S1600536810035051/zs2058Isup2.hkl

Additional supplementary materials:  crystallographic information; 3D view; checkCIF report

## Figures and Tables

**Table 1:** Hydrogen-bond geometry (Å, °)

*D*—H⋯*A*	*D*—H	H⋯*A*	*D*⋯*A*	*D*—H⋯*A*
N5—H5⋯O2*A*^i^	0.86	1.92	2.704 (3)	151
N6—H6*A*⋯Cl3	0.86	2.62	3.417 (3)	155
N6—H6*B*⋯O2*A*^i^	0.86	2.47	3.116 (3)	132
N6—H6*B*⋯Cl3^i^	0.86	2.80	3.383 (2)	127
N5*A*—H5*A*⋯O2^ii^	0.86	1.87	2.692 (2)	158
N6*A*—H6*A*1⋯Cl2	0.86	2.51	3.336 (3)	161
N6*A*—H6*A*2⋯Cl3^iii^	0.86	2.73	3.430 (2)	139

Symmetry codes: (i) 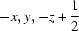; (ii) 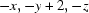; (iii) 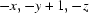.

**Fig. 1.**
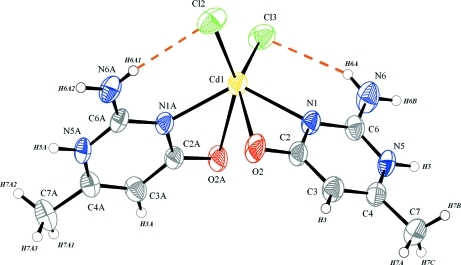
A view of the title compound, showing the atom numbering scheme. with 50% probability displacement ellipsoids.

**Fig. 2.**
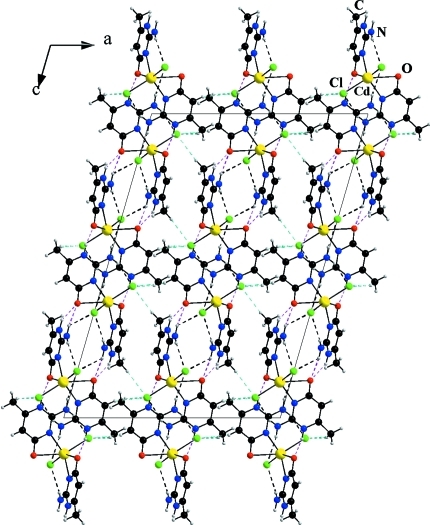
The packing of (I) viewed down the b axis. Hydrogen bonds are denoted by dashed lines.

**Fig. 3.**
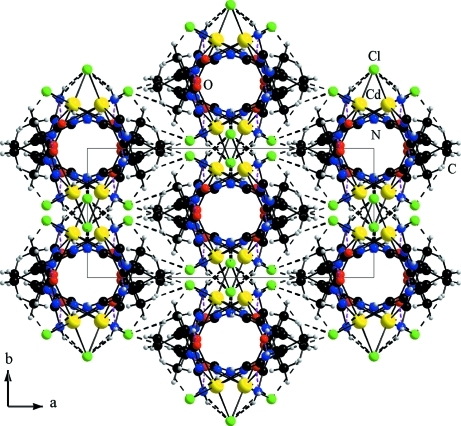
The packing of (I) viewed down the c axis. Hydrogen bonds are denoted by dashed lines.

## supplementary crystallographic information

### Comment

Open framework crystalline solids containing micro or mesoporisity have been studied extensively in recent years, due to their intriguing structures and considerable technological importance in magnetic, luminescent, porous and catalytic materials (Yaghi, *et al.*, 2003; Kitagawa, *et al.*, 2004). In this work a new member of this family is presented, the title complex involving CdCl_2_ and the ligand 2-amino-4-hydroxy-6-methylpyrimidine, [Cd(C_5_H_7_N_3_O)_2_Cl_2_], (I) which was obtained during our studies on the preparation of new organometallic materials (Kaabi,*et al.*, 2010).

In the atomic arrangement of the title compound, the distorted octahedral Cd environment comprises two chloride donor atoms and two N and two O donor atoms from two bidentate chelate organic ligands (Fig. 1). The bond distances around the Cd atom [Cd—N, 2.261 (2), 2.286 (2) Å; Cd—O, 2.624 (2), 2.692 (2) Å; Cd—Cl, 2.4674 (6), 2.4893 (7) Å] are normal (Min *et al.*, 2009; Qing-Yan & Li, 2005; Moloto *et al.*, 2003). The octahedra have intramolecular N—H···Cl hydrogen bonds and are interconnected by a set of N—H···Cl and N—H···O hydrogen bonds (Table 1) leading to the formation of a three-dimensional network structure (Fig. 2). Among the hydrogen bonds, one is three-centred [N6—H6B···(O2A,Cl3)]. The overall packing pattern, presented in Fig. 3, shows that the different components of the title material are arranged so as to create pores extending along the *c* axis and located at (0, 0, 0) and (1/2, 1/2, 0). Thus, this organic-inorganic hybrid open framework material could have potential application as a molecular sieve. An examination of the organic moiety features shows that the bond distances for C2—O2 [1.248 (3) Å] and C2A—O2A [1.255 (3) Å] clearly indicate two double bonds. This allows us to confirm that the first step of the preparation of the title compound consists of the transformation of the 2-amino-6-methyl-4-pyrimidinol into 2-amino-6-methylpyrimidin-4-(1*H*)-one. However, the present investigation clearly shows that the N6—C6 [1.332 (3) Å] and N6A—C6A [1.324 (3) Å] distances are approximately equal to that of a C=N double bond length, indicating that N3 and N6 nitrogen atoms of the amino group are probably in an *sp*^2^ hybridization. These bond length features are consistent with imino resonance and suggest a large contribution from it to the stability of the title compound.

### Experimental

A solution of CdCl_2_ (37 mg, 0.2 mmol) in water (6 ml) was added dropwise to a solution of 2-amino-4-hydroxy-6-methylpyrimidine (50 mg, 0.4 mmol) in ethanol (6 ml). After stirring for 30 min, the mixture was filtered and the resultant solution allowed to evaporate at room temperature. Crystals of the title compound, which remained stable under normal conditions of temperature and humidity, were isolated after several days and subjected to X-ray diffraction analysis (yield 58%).

### Refinement

All H atoms were located in a difference Fourier synthesis but were placed in calculated positions and allowed to ride on their parent atoms, with C—H_aromatic_ = 0.93 Å, C—H_methyl_ = 0.96 Å and N—H = 0.86 Å, and with U_iso_ = 1.2–1.5U</i_eq_(C).

### Crystal data

**Table:**

[CdCl_2_(C_5_H_7_N_3_O)_2_]	*F*(000) = 1712
*M**_r_* = 433.57	*D*_x_ = 1.793 Mg m^−^^3^
Monoclinic, *C*2/*c*	Mo *K*α radiation, λ = 0.71073 Å
Hall symbol: -C 2yc	Cell parameters from 9775 reflections
*a* = 17.4204 (5) Å	θ = 3.0–29.3°
*b* = 7.5467 (2) Å	µ = 1.70 mm^−^^1^
*c* = 25.4422 (6) Å	*T* = 293 K
β = 106.1333 (11)°	Flat prism, colourless
*V* = 3213.07 (15) Å^3^	0.32 × 0.20 × 0.13 mm
*Z* = 8	

### Data collection

**Table:**

Bruker APEXII CCD area-detector diffractometer	4494 independent reflections
Radiation source: fine-focus sealed tube	3924 reflections with *I* > 2σ(*I*)
graphite	*R*_int_ = 0.033
φ and ω scans	θ_max_ = 29.5°, θ_min_ = 1.7°
Absorption correction: multi-scan (*SADABS*; Sheldrick, 2003)	*h* = −24→24
*T*_min_ = 0.676, *T*_max_ = 0.801	*k* = −10→10
41891 measured reflections	*l* = −35→35

### Refinement

**Table:**

Refinement on *F*^2^	Primary atom site location: structure-invariant direct methods
Least-squares matrix: full	Secondary atom site location: difference Fourier map
*R*[*F*^2^ > 2σ(*F*^2^)] = 0.023	Hydrogen site location: inferred from neighbouring sites
*wR*(*F*^2^) = 0.108	H-atom parameters constrained
*S* = 1.26	*w* = 1/[σ^2^(*F*_o_^2^) + (0.0634*P*)^2^ + 0.0986*P*] where *P* = (*F*_o_^2^ + 2*F*_c_^2^)/3
4494 reflections	(Δ/σ)_max_ < 0.001
190 parameters	Δρ_max_ = 0.74 e Å^−^^3^
0 restraints	Δρ_min_ = −1.06 e Å^−^^3^

### Special details

**Table:**

Geometry. All e.s.d.'s (except the e.s.d. in the dihedral angle between two l.s. planes) are estimated using the full covariance matrix. The cell e.s.d.'s are taken into account individually in the estimation of e.s.d.'s in distances, angles and torsion angles; correlations between e.s.d.'s in cell parameters are only used when they are defined by crystal symmetry. An approximate (isotropic) treatment of cell e.s.d.'s is used for estimating e.s.d.'s involving l.s. planes.
Refinement. Refinement of *F*^2^ against ALL reflections. The weighted *R*-factor *wR* and goodness of fit *S* are based on *F*^2^, conventional *R*-factors *R* are based on *F*, with *F* set to zero for negative *F*^2^. The threshold expression of *F*^2^ > σ(*F*^2^) is used only for calculating *R*-factors(gt) *etc*. and is not relevant to the choice of reflections for refinement. *R*-factors based on *F*^2^ are statistically about twice as large as those based on *F*, and *R*- factors based on ALL data will be even larger. 5 reflections were affected by the beamstop.

### Fractional atomic coordinates and isotropic or equivalent isotropic displacement parameters (Å^2^)

**Table:**

	*x*	*y*	*z*	*U*_iso_*/*U*_eq_	
Cd1	0.049831 (10)	0.66207 (2)	0.118222 (6)	0.03647 (9)	
Cl2	0.14243 (4)	0.55912 (10)	0.06682 (3)	0.05302 (18)	
Cl3	0.00058 (5)	0.38970 (10)	0.15353 (3)	0.05174 (18)	
O2	0.11698 (14)	0.9762 (3)	0.13204 (7)	0.0528 (5)	
N1	0.10788 (13)	0.7995 (3)	0.19965 (8)	0.0359 (4)	
N5	0.13746 (13)	0.9069 (3)	0.28892 (8)	0.0403 (5)	
H5	0.1385	0.8886	0.3225	0.048*	
N6	0.09525 (17)	0.6185 (3)	0.26931 (9)	0.0538 (6)	
H6A	0.0802	0.5332	0.2463	0.065*	
H6B	0.0986	0.6023	0.3033	0.065*	
C2	0.12795 (15)	0.9609 (3)	0.18243 (9)	0.0383 (5)	
C3	0.15860 (17)	1.0967 (3)	0.22184 (10)	0.0432 (6)	
H3	0.1770	1.2029	0.2113	0.052*	
C4	0.16041 (15)	1.0686 (3)	0.27419 (10)	0.0381 (5)	
C6	0.11331 (14)	0.7758 (4)	0.25216 (9)	0.0365 (5)	
C7	0.1859 (2)	1.2017 (4)	0.31928 (12)	0.0556 (7)	
H7A	0.1992	1.3112	0.3047	0.083*	
H7B	0.2319	1.1580	0.3465	0.083*	
H7C	0.1431	1.2213	0.3356	0.083*	
O2A	−0.08881 (13)	0.8086 (3)	0.12328 (7)	0.0480 (5)	
N1A	−0.04596 (12)	0.7847 (3)	0.04894 (7)	0.0329 (4)	
N5A	−0.12319 (13)	0.8922 (3)	−0.03495 (8)	0.0358 (4)	
H5A	−0.1284	0.9100	−0.0692	0.043*	
N6A	0.00074 (14)	0.7722 (3)	−0.02733 (9)	0.0439 (5)	
H6A1	0.0447	0.7261	−0.0080	0.053*	
H6A2	−0.0063	0.7913	−0.0617	0.053*	
C2A	−0.10327 (16)	0.8387 (3)	0.07295 (10)	0.0352 (5)	
C3A	−0.17409 (16)	0.9202 (4)	0.04057 (10)	0.0416 (5)	
H3A	−0.2138	0.9565	0.0562	0.050*	
C4A	−0.18262 (14)	0.9439 (3)	−0.01337 (10)	0.0382 (5)	
C6A	−0.05619 (15)	0.8137 (3)	−0.00417 (9)	0.0334 (5)	
C7A	−0.25489 (18)	1.0267 (4)	−0.05236 (12)	0.0544 (7)	
H7A1	−0.2961	1.0418	−0.0343	0.082*	
H7A2	−0.2740	0.9512	−0.0836	0.082*	
H7A3	−0.2407	1.1401	−0.0640	0.082*	

### Atomic displacement parameters (Å^2^)

**Table:**

	*U*^11^	*U*^22^	*U*^33^	*U*^12^	*U*^13^	*U*^23^
Cd1	0.03843 (13)	0.04544 (14)	0.02572 (12)	0.00425 (7)	0.00919 (8)	−0.00182 (6)
Cl2	0.0401 (3)	0.0646 (4)	0.0606 (4)	−0.0029 (3)	0.0243 (3)	−0.0193 (3)
Cl3	0.0707 (5)	0.0453 (3)	0.0463 (4)	−0.0026 (3)	0.0282 (4)	−0.0019 (3)
O2	0.0827 (15)	0.0568 (11)	0.0220 (8)	−0.0044 (10)	0.0196 (9)	0.0058 (7)
N1	0.0453 (12)	0.0419 (10)	0.0224 (9)	−0.0027 (9)	0.0125 (8)	0.0001 (7)
N5	0.0474 (12)	0.0558 (12)	0.0186 (8)	−0.0015 (10)	0.0108 (8)	−0.0001 (8)
N6	0.0727 (18)	0.0589 (13)	0.0322 (12)	−0.0167 (13)	0.0189 (12)	0.0060 (10)
C2	0.0455 (13)	0.0467 (13)	0.0234 (10)	0.0014 (11)	0.0110 (10)	0.0034 (9)
C3	0.0525 (16)	0.0434 (13)	0.0336 (12)	−0.0048 (12)	0.0121 (11)	0.0021 (10)
C4	0.0347 (12)	0.0483 (13)	0.0298 (11)	0.0013 (10)	0.0066 (9)	−0.0026 (9)
C6	0.0379 (12)	0.0479 (12)	0.0262 (11)	0.0005 (10)	0.0128 (9)	0.0057 (10)
C7	0.0622 (19)	0.0615 (16)	0.0383 (14)	0.0043 (15)	0.0059 (13)	−0.0127 (13)
O2A	0.0588 (12)	0.0672 (12)	0.0216 (8)	0.0185 (10)	0.0173 (8)	0.0067 (7)
N1A	0.0368 (10)	0.0419 (10)	0.0218 (9)	0.0025 (8)	0.0112 (8)	0.0004 (7)
N5A	0.0423 (11)	0.0450 (10)	0.0194 (8)	−0.0070 (9)	0.0076 (8)	0.0030 (8)
N6A	0.0480 (12)	0.0592 (13)	0.0298 (10)	−0.0047 (11)	0.0199 (9)	−0.0062 (10)
C2A	0.0417 (13)	0.0421 (12)	0.0239 (11)	0.0031 (9)	0.0127 (10)	0.0028 (8)
C3A	0.0399 (13)	0.0533 (14)	0.0346 (12)	0.0079 (11)	0.0156 (10)	0.0093 (11)
C4A	0.0364 (12)	0.0440 (12)	0.0318 (12)	−0.0039 (10)	0.0055 (10)	0.0080 (9)
C6A	0.0402 (13)	0.0391 (11)	0.0222 (10)	−0.0098 (9)	0.0110 (9)	−0.0044 (8)
C7A	0.0479 (16)	0.0656 (18)	0.0435 (15)	0.0020 (14)	0.0023 (13)	0.0191 (13)

### Geometric parameters (Å, °)

**Table:**

Cd1—N1A	2.261 (2)	C7—H7A	0.9600
Cd1—N1	2.286 (2)	C7—H7B	0.9600
Cd1—Cl2	2.4674 (6)	C7—H7C	0.9600
Cd1—Cl3	2.4893 (7)	O2A—C2A	1.255 (3)
Cd1—O2	2.624 (2)	N1A—C6A	1.331 (3)
Cd1—O2A	2.692 (2)	N1A—C2A	1.369 (3)
O2—C2	1.248 (3)	N5A—C6A	1.348 (3)
N1—C6	1.325 (3)	N5A—C4A	1.357 (3)
N1—C2	1.372 (3)	N5A—H5A	0.8600
N5—C6	1.346 (3)	N6A—C6A	1.324 (3)
N5—C4	1.369 (3)	N6A—H6A1	0.8600
N5—H5	0.8600	N6A—H6A2	0.8600
N6—C6	1.332 (3)	C2A—C3A	1.419 (4)
N6—H6A	0.8600	C3A—C4A	1.351 (3)
N6—H6B	0.8600	C3A—H3A	0.9300
C2—C3	1.429 (3)	C4A—C7A	1.505 (3)
C3—C4	1.340 (3)	C7A—H7A1	0.9600
C3—H3	0.9300	C7A—H7A2	0.9600
C4—C7	1.496 (4)	C7A—H7A3	0.9600
			
Cl2—Cd1—Cl3	105.84 (3)	N1—C6—N5	121.5 (2)
Cl2—Cd1—O2	91.30 (5)	N6—C6—N5	118.9 (2)
Cl2—Cd1—O2A	151.90 (4)	C4—C7—H7A	109.5
Cl2—Cd1—N1	115.69 (6)	C4—C7—H7B	109.5
Cl2—Cd1—N1A	99.51 (5)	H7A—C7—H7B	109.5
Cl3—Cd1—O2	152.05 (4)	C4—C7—H7C	109.5
Cl3—Cd1—O2A	85.31 (5)	H7A—C7—H7C	109.5
Cl3—Cd1—N1	99.07 (6)	H7B—C7—H7C	109.5
Cl3—Cd1—N1A	111.44 (6)	C6A—N1A—C2A	119.7 (2)
O2—Cd1—O2A	89.71 (7)	C6A—N1A—Cd1	136.08 (17)
O2—Cd1—N1	53.14 (7)	C2A—N1A—Cd1	104.22 (14)
O2—Cd1—N1A	86.54 (7)	C6A—N5A—C4A	121.7 (2)
O2A—Cd1—N1	86.97 (7)	C6A—N5A—H5A	119.1
O2A—Cd1—N1A	52.52 (6)	C4A—N5A—H5A	119.1
N1—Cd1—N1A	124.44 (8)	C6A—N6A—H6A1	120.0
C2—O2—Cd1	89.39 (15)	C6A—N6A—H6A2	120.0
C6—N1—C2	119.2 (2)	H6A1—N6A—H6A2	120.0
C6—N1—Cd1	137.93 (17)	O2A—C2A—N1A	115.9 (2)
C2—N1—Cd1	101.61 (14)	O2A—C2A—C3A	124.5 (2)
C6—N5—C4	121.6 (2)	N1A—C2A—C3A	119.6 (2)
C6—N5—H5	119.2	C4A—C3A—C2A	118.7 (2)
C4—N5—H5	119.2	C4A—C3A—H3A	120.7
C6—N6—H6A	120.0	C2A—C3A—H3A	120.7
C6—N6—H6B	120.0	C3A—C4A—N5A	119.5 (2)
H6A—N6—H6B	120.0	C3A—C4A—C7A	124.1 (3)
O2—C2—N1	115.5 (2)	N5A—C4A—C7A	116.4 (2)
O2—C2—C3	125.1 (2)	N6A—C6A—N1A	120.5 (2)
N1—C2—C3	119.4 (2)	N6A—C6A—N5A	118.7 (2)
C4—C3—C2	119.0 (2)	N1A—C6A—N5A	120.8 (2)
C4—C3—H3	120.5	C4A—C7A—H7A1	109.5
C2—C3—H3	120.5	C4A—C7A—H7A2	109.5
C3—C4—N5	119.0 (2)	H7A1—C7A—H7A2	109.5
C3—C4—C7	125.1 (3)	C4A—C7A—H7A3	109.5
N5—C4—C7	115.8 (2)	H7A1—C7A—H7A3	109.5
N1—C6—N6	119.5 (2)	H7A2—C7A—H7A3	109.5
			
N1A—Cd1—O2—C2	135.12 (17)	Cd1—N1—C6—N5	−164.08 (19)
N1—Cd1—O2—C2	−3.77 (15)	C4—N5—C6—N1	−2.5 (4)
Cl2—Cd1—O2—C2	−125.43 (16)	C4—N5—C6—N6	176.9 (2)
Cl3—Cd1—O2—C2	3.3 (2)	N1—Cd1—N1A—C6A	126.3 (2)
N1A—Cd1—N1—C6	117.1 (3)	Cl2—Cd1—N1A—C6A	−4.0 (2)
Cl2—Cd1—N1—C6	−119.4 (2)	Cl3—Cd1—N1A—C6A	−115.3 (2)
Cl3—Cd1—N1—C6	−6.8 (3)	O2—Cd1—N1A—C6A	86.7 (2)
O2—Cd1—N1—C6	169.8 (3)	N1—Cd1—N1A—C2A	−52.71 (18)
N1A—Cd1—N1—C2	−49.21 (18)	Cl2—Cd1—N1A—C2A	176.92 (14)
Cl2—Cd1—N1—C2	74.29 (16)	Cl3—Cd1—N1A—C2A	65.63 (15)
Cl3—Cd1—N1—C2	−173.16 (14)	O2—Cd1—N1A—C2A	−92.33 (15)
O2—Cd1—N1—C2	3.50 (14)	C6A—N1A—C2A—O2A	−179.1 (2)
Cd1—O2—C2—N1	5.6 (2)	Cd1—N1A—C2A—O2A	0.1 (3)
Cd1—O2—C2—C3	−174.0 (3)	C6A—N1A—C2A—C3A	1.9 (3)
C6—N1—C2—O2	−176.1 (2)	Cd1—N1A—C2A—C3A	−178.86 (19)
Cd1—N1—C2—O2	−6.5 (3)	O2A—C2A—C3A—C4A	−179.5 (3)
C6—N1—C2—C3	3.6 (4)	N1A—C2A—C3A—C4A	−0.7 (4)
Cd1—N1—C2—C3	173.1 (2)	C2A—C3A—C4A—N5A	−1.0 (4)
O2—C2—C3—C4	173.8 (3)	C2A—C3A—C4A—C7A	179.4 (2)
N1—C2—C3—C4	−5.8 (4)	C6A—N5A—C4A—C3A	1.5 (4)
C2—C3—C4—N5	4.0 (4)	C6A—N5A—C4A—C7A	−178.9 (2)
C2—C3—C4—C7	−176.0 (3)	C2A—N1A—C6A—N6A	175.9 (2)
C6—N5—C4—C3	0.1 (4)	Cd1—N1A—C6A—N6A	−3.0 (4)
C6—N5—C4—C7	−179.9 (2)	C2A—N1A—C6A—N5A	−1.5 (3)
C2—N1—C6—N6	−178.8 (2)	Cd1—N1A—C6A—N5A	179.61 (17)
Cd1—N1—C6—N6	16.5 (4)	C4A—N5A—C6A—N6A	−177.6 (2)
C2—N1—C6—N5	0.5 (4)	C4A—N5A—C6A—N1A	−0.3 (3)

### Hydrogen-bond geometry (Å, °)

**Table:**

*D*—H···*A*	*D*—H	H···*A*	*D*···*A*	*D*—H···*A*
N5—H5···O2A^i^	0.86	1.92	2.704 (3)	151
N6—H6A···Cl3	0.86	2.62	3.417 (3)	155
N6—H6B···O2A^i^	0.86	2.47	3.116 (3)	132
N6—H6B···Cl3^i^	0.86	2.80	3.383 (2)	127
N5A—H5A···O2^ii^	0.86	1.87	2.692 (2)	158
N6A—H6A1···Cl2	0.86	2.51	3.336 (3)	161
N6A—H6A2···Cl3^iii^	0.86	2.73	3.430 (2)	139

Symmetry codes: (i) −*x*, *y*, −*z*+1/2; (ii) −*x*, −*y*+2, −*z*; (iii) −*x*, −*y*+1, −*z*.
